# Supplementary material for: Bacterial Community Structure of Pinus Thunbergii Naturally Infected by the Nematode Bursaphelenchus Xylophilus
Source: Microorganisms. 2020 Feb 23;8(2):307. doi: 10.3390/microorganisms8020307 (PMC7074913; doi:10.3390/microorganisms8020307)
Supplement: Supplementary file 1 [file microorganisms-08-00307-s001.pdf]

Table 1. The bacterial community diversity estimators in needle, root and soil.

Samples		Sobs	Chao 1	Shannon	Shannoneven
Needle	diseased	172 ± 10	255.91 ± 9.18	3.62 ± 0.11	0.70 ± 0.01
	healthy	151 ± 8	227.55 ± 27.47	3.51 ± 0.19	0.70 ± 0.03
Root	diseased	685 ± 75	1322.18 ± 185.04	5.55 ± 0.22	0.86 ± 0.02
	healthy	657 ± 47	1279.24 ± 133.85	5.42 ± 0.08	0.84 ± 0.01
Soil	diseased	956 ± 60	2132.93 ± 90.83	6.14 ± 0.14	0.89 ± 0.01
	healthy	924 ± 14	1978.91 ± 39.38	6.11 ± 0.07	0.90 ± 0.01

The values are shown as means ± standard deviation (n=9, except for soil n=27).

Table 2. The relative abundance of phylum between healthy (H) and diseased (D) trees in Root (R), Needle (N) and Soil (S).

taxon	Total	Needle		Root		Soil	
		DN	HN	DR	HR	DS	HS
Proteobacteria	41.9 ± 4.5%	79.1 ± 3.2%	73.4 ± 4.8%	44.5 ± 6.5%*	35.4 ± 2.4%	36.8 ± 1.9%	37.0 ± 0.4%
Actinobacteria	29.0 ± 3.7%	3.1 ± 1.2%	3.4 ± 0.9%	36.1 ± 5.8%	36.6 ± 4.4%	31.5 ± 5.2%	28.2 ± 3.2%
Acidobacteria	12.2 ± 1.4%	1.9 ± 0.5%	3.9 ± 1.2%	7.0 ± 0.4%	11.3 ± 1.5%	13.8 ± 1.7%	16.5 ± 2.7%
Bacteroidetes	4.8 ± 1.0%	11.7 ± 1.0%	10.6 ± 3.3%	3.5 ± 0.4%	2.4 ± 0.3%	4.7 ± 1.5%	4.3 ± 0.8%
Bacteria_unclassified	4.6 ± 0.6%	3.0 ± 1.7%	6.3 ± 2.2%	2.2 ± 0.3%	4.7 ± 1.3%	5.0 ± 0.4%	5.5 ± 0.5%
Planctomycetes	2.1 ± 0.3%	1.0 ± 0.2%	1.3 ± 0.4%	1.1 ± 0.2%	3.7 ± 0.6%	2.0 ± 0.4%	2.4 ± 0.4%
Verrucomicrobia	1.8 ± 0.2%			1.2 ± 0.4%	1.9 ± 0.6%	2.2 ± 0.4%	2.1 ± 0.3%
Candidatus_Saccharibacteria	1.4 ± 0.2%			1.4 ± 0.3%	0.8 ± 0.1%	1.7 ± 0.2%	1.7 ± 0.1%
Chloroflexi	1.2 ± 0.2%			0.9 ± 0.2%	1.9 ± 0.2%	1.4 ± 0.2%	1.3 ± 0.3%
Gemmatimonadetes	0.6 ± 0.1%			0.3 ± 0.1%	0.6 ± 0.2%	0.8 ± 0.2%	0.7 ± 0.2%
Firmicutes	0.5 ± 0.2%	0.2 ± 0.1%	1.0 ± 0.1%	1.8 ± 0.4%*	0.6 ± 0.1%	0.2 ± 0.1%	0.3 ± 0.1%

The values are shown as means ± standard deviation (n=9, except for soil n=27). * $P < 0.05$, significant difference in global Kruskal-Wallis test.

Table 3. The relative abundance of Order and Genus between healthy (H) and diseased (D) trees in Root (R), Needle (N) and Soil (S).

	taxon	Total	Needle		Root		Soil	
			DN	HN	DR	HR	DS	HS
Order	**Actinomycetales**	16.5 ± 2.4%	3.0 ± 1.2%	3.3 ± 0.9%	24.9 ± 5.2%	23.9 ± 4.2%	15.9 ± 1.2%	14.9 ± 2.3%
	Rhizobiales	13.9 ± 3.1%	26.7 ± 0.1%	45.7 ± 1.1%*	6.3 ± 1.1%	14.7 ± 0.9%*	13.7 ± 0.5%	12.7 ± 1.1%
	Rhodospirillales	6.1 ± 0.4%	3.0 ± 0.3%	4.9 ± 0.8%	5.7 ± 0.3%	5.6 ± 0.3%	6.4 ± 0.3%	7.0 ± 1.1%
	Burkholderiales	5.0 ± 1.6%	18.7 ± 4.7%a	2.6 ± 0.6%	9.1 ± 2.1%*	1.2 ± 0.2%	3.1 ± 0.2%	2.8 ± 0.1%
	Gaiellales	4.0 ± 0.6%			2.6 ± 0.4%	2.6 ± 0.5%	5.9 ± 0.6%	4.4 ± 1.0%
	Sphingomonadales	3.5 ± 1.4%	13.8 ± 0.6%	12.7 ± 1.0%	6.3 ± 3.0%*	0.5 ± 0.1%	1.7 ± 0.1%	2.0 ± 1.3%
	Gp6	3.0 ± 0.6%			0.7 ± 0.1%	1.1 ± 0.1%	4.0 ± 0.3%	4.5 ± 1.9%
Genus	Gaiella	4.0 ± 0.6%			2.6 ± 0.4%	2.6 ± 0.5%	5.9 ± 1.5%	4.4 ± 1.0%
	Burkholderia	2.3 ± 0.7%			7.8 ± 1.9%*	0.7 ± 0.2%	1.7 ± 0.4%	1.6 ± 0.2%
	Sphingomonas	1.7 ± 1.1%	11.1 ± 0.9%	8.9 ± 0.2%	1.6 ± 0.9%*	0.2 ± 0.1%	0.5 ± 0.1%	0.7 ± 0.2%
	Massilia	1.4 ± 0.2%	16.2 ± 4.9%*	1.4 ± 0.5%				
	Mycobacterium	1.2 ± 0.2%			1.7 ± 0.5%	1.1 ± 0.1%	1.5 ± 0.2%	1.2 ± 0.2%
	Actinospica	1.1 ± 0.7%			4.1 ± 2.0%	4.2 ± 2.3%	0.1 ± 0.1%	0.1 ± 0.1%
	Methylobacterium	1.0 ± 0.1%	9.9 ± 0.6%	13.1 ± 3.2%				

The values are shown as means ± standard deviation (n=9, except for soil n=27). * $P < 0.05$, significant difference in global Kruskal-Wallis test.
